# Effects of laryngeal mask ventilation on postoperative atelectasis in children undergoing day surgery: a randomized controlled trial

**DOI:** 10.1186/s12871-023-02327-2

**Published:** 2023-11-06

**Authors:** Weiwei Cai, Wei Gu, Huanhuan Ni, Longde Zhao, Shan Zhong, Wei Wang

**Affiliations:** 1 Department of Anesthesiology, Children’s Hospital of Nanjing Medical University, Nanjing, 210000 China; 2 Department of Statistics, Children’s Hospital of Nanjing Medical University, Nanjing, 210000 China; 3 Department of Anesthesiology, Children’s Hospital of Fudan University, ShangHai, 201102 China

**Keywords:** Laryngeal mask, Day surgery, Pulmonary atelectasis, Lung ultrasound mechanical ventilation

## Abstract

**Background:**

To compare the effects of laryngeal mask mechanical ventilation and preserved spontaneous breathing on postoperative atelectasis in children undergoing day surgery.

**Methods:**

Children aged 3–7 who underwent elective day surgery were randomly divided into a spontaneous breathing group (*n* = 23) and a mechanical ventilation group (*n* = 23). All children enrolled in this trial used the same anesthesia induction protocol, the incidence and severity of atelectasis before induction and after operation were collected. In addition, the baseline data, intraoperative vital signs, ventilator parameters and whether there were complications such as reflux and aspiration were also collected. SPSS was used to calculate whether there was a statistical difference between these indicators.

**Results:**

The incidence of atelectasis in the spontaneous breathing group was 91.30%, and 39.13% in the mechanical ventilation group, and the difference was statistically significant (*P* = 0.001). There was a statistically significant difference in carbon dioxide (*P* < 0.05), and the severity of postoperative atelectasis in the mechanical ventilation group was lower than that in the spontaneous breathing group (*P* < 0.05). In addition, there were no significant differences in the vital signs and baseline data of the patients (*P* > 0.05).

**Conclusion:**

Laryngeal mask mechanical ventilation can reduce the incidence and severity of postoperative atelectasis in children undergoing day surgery, and we didn’t encounter any complications such as reflux and aspiration in children during the perioperative period, so mechanical ventilation was recommended to be used for airway management.

**Trial registration:**

The clinical trial was registered retrospectively at the Chinese Clinical Trial Registry. (https://www.chictr.org.cn. Registration number ChiCTR2300071396, Weiwei Cai, 15 May 2023).

## Introduction

Atelectasis is a common perioperative complication. It has been reported [1] that the incidence of atelectasis in children with laryngeal mask general anesthesia ranges from 68% to 100%, which indicating that the incidence of pulmonary atelectasis in patients undergoing laryngeal mask general anesthesia is high. In addition, postoperative atelectasis is not easy to recover in a short term and can last for several days. Even minimal atelectasis can lead to postoperative complications such as hypoxemia, pneumonia, and ARDS [2].

Day surgery refers to operations that require the participation of anesthesiologists within 24 h from admission to discharge. These children were hospitalised for a shorter period of time for observation and are prone to complications such as pulmonary infection after discharge due to the presence of atelectasis. Therefore, it is important to pay more attention to postoperative atelectasis in children who underwent day surgery.

Laryngeal mask is a supraglottic airway tool, which has been commonly used in clinical because of its non-invasiveness, less damage, and easy to insert. Now, the use of laryngeal mask instead of tracheal intubation for airway management has been achieved in day surgery, therefore, how to performe a respiratory management with a laryngeal mask is particularly important.

It was reported that muscle relaxants may lead to an increase in postoperative pulmonary complications, and laryngeal mask retention of spontaneous breathing can reduce the incidence of reflux aspiration, so laryngeal mask retention of spontaneous breathing has become a common method of respiratory management for children [3–9]. In addition, mechanical ventilation is also a commonly used method of airway management in clinical practice. However, which method is better is still unclear, only a few literatures have analyzed from the perspective of perioperative breathing-related parameter, and there is no research focused on the impact of these two methods from the perspective of postoperative atelectasis, therefore the aim of this study was to evaluate the effect of laryngeal mask ventilation on postoperative pulmonary atelectasis in children by lung ultrasound, and to provide a better ventilation method for doctors.

## Materials and methods

This study was performed at the Children’s Hospital of Nanjing Medical University from April to August 2022 and was approved by the Ethics Committee of the Children’s Hospital of Nanjing Medical University (approval number: 202112124–1). Inclusion criteria: 1) Children aged 3 ~ 7 years, ASA class I or II, undergoing day surgery, with cryptorchidism and indirect inguinal hernia as the disease type, and open surgery as the surgical method, 2) Operation time < 2 h. Exclusion criteria: 1) preoperative respiratory infection, 2) severe cardiopulmonary disease, 3) overweight (more than 20% of standard body weight), 4) neuromuscular disease, 5) abnormal preoperative chest radiograph or chest CT, inflammatory parameters, 6) the child’s guardian did not agree to participate in this trial, 7) preoperative lung ultrasound suggested the presence of pulmonary atelectasis, 8) after removal of the laryngeal mask, the patient’s SpO2 < 90%.

### Randomization and blinding

Group allocation was concealed in sequentially numbered, opaque, sealed envelopes that were opened by trained study personnel. Each envelope contained the group allocation and instructions for the attending anaesthesiologist. Weiwei Cai generated the random allocation sequence, Huanhuan Ni enrolled participants, and Longde Zhao assigned participants to interventions. Weiwei Cai was blinded after assignment to interventions and performed the second lung ultrasound examination. Finally 46 children were randomly divided into spontaneous breathing group (*n* = 23) and mechanical ventilation group (*n* = 23).

### Anesthesia

An intravenous route was established before the patient was brought to the operating room. An electrocardiogram, pulse oxygen saturation, blood pressure, end-tidal carbon dioxide, respiratory rate and body temperature were monitored after entering the operating room. A standard anesthetic protocol was used for all patients, including midazolam 0.05 mg-kg^−1^, propofol 2-3 mg-kg^−1^, sufentanil 0.3ug-kg^−1^. And anesthesia was maintained using sevoflurane 2–3% and propofol 5 mg-kg^−1^ -h^−1^. Intraoperative BIS monitoring was used to maintain the depth of anesthesia between 40 and 60. Sufentanil was given slowly intravenously during induction to ensure the presence of spontaneous breathing. After 2–3 min of administration, the child’s mandible was held up and if the child did not respond, a laryngeal mask was inserted (choose the appropriate type of mask according to the child’s weight). The inhalation oxygen concentration during induction and intraoperative maintenance was 60%. After the insertion of the laryngeal mask, spontaneous ventilation was maintained in the spontaneous breathing group and controlled ventilation was performed in the mechanical ventilation group using the PCV mode. The driving pressure was set to ensure that the child’s exhaled tidal volume was approximately 6–8 ml/kg, the respiratory rate was set to 20 breaths/min, and the inspiratory-to-expiratory ratio was set to 1:2. After surgery, the laryngeal mask was removed immediately in the spontaneous breathing group. In the mechanical ventilation group, the ventilator mode was adjusted to manual breathing at the end of surgery, the APL valve was set to 0 cmH2O, and the inhale oxygen concentration was 60%. If the patient has SpO2 < 90%, the ventilator will be switched to PCV mode, the intraoperative driving pressure of this patient was set to ensure tidal volume reach to 6-8 ml/kg, and a respiratory rate of 20 breaths/minute was also set. When the patient’s SpO2 > 95%, the ventilator mode would be switched to manual ventilation again, and the laryngeal mask would be removed if the child’s spontaneous breathing had recovered. After removal of the laryngeal mask, 60% oxygen was inhaled in both two groups. After surgery, if children’s FLACC score ≥ 4, we will give 0.1–0.2ug-kg^−1^ sufentanil intravenously to alleviate pain.

### Lung ultrasound examination

**Fig. 1:**
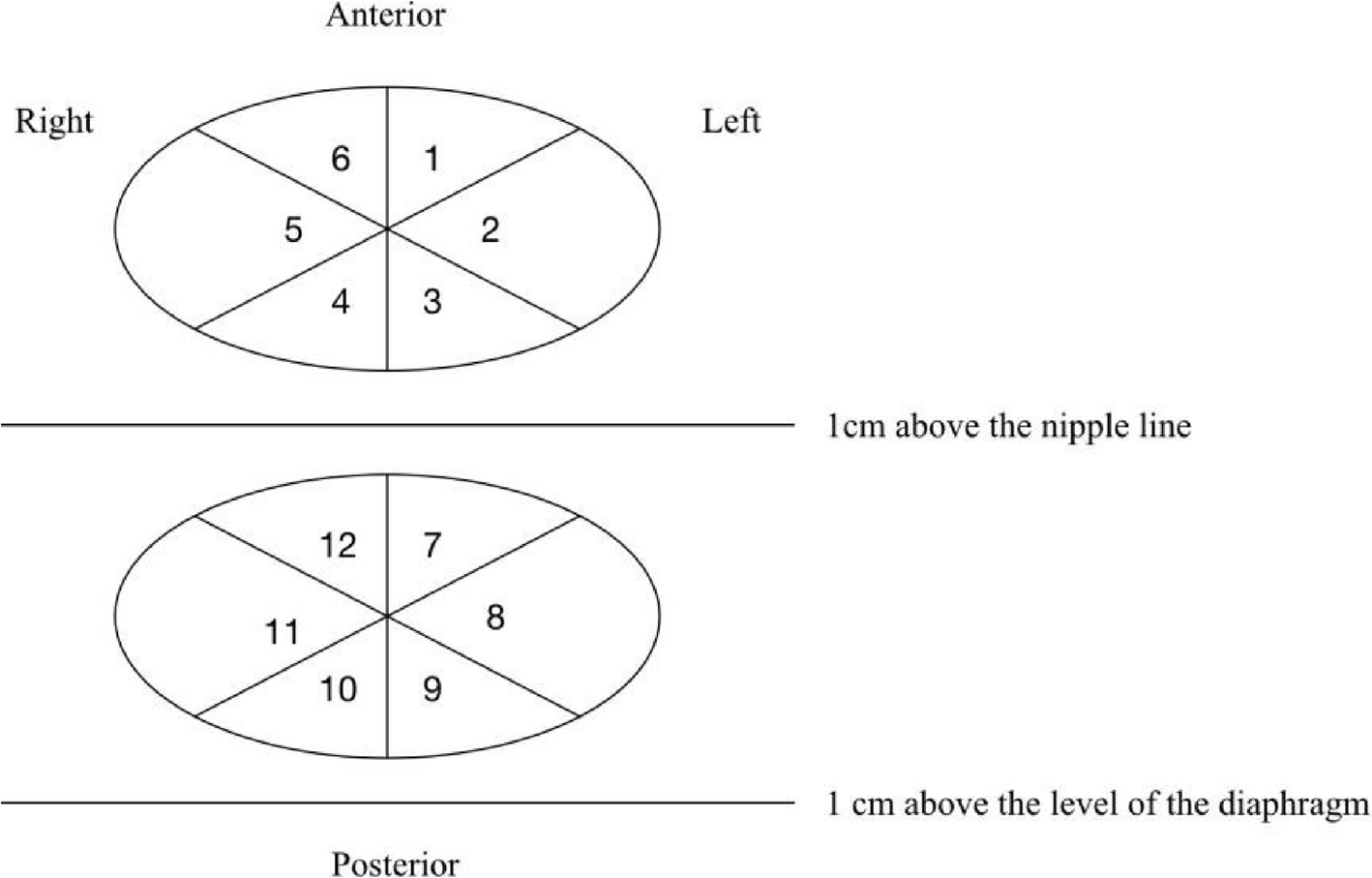
Partition of lung ultrasound

Lung ultrasound was performed twice before the induction of anesthesia and 20 min after the removal of the laryngeal mask. The lung ultrasound was performed by a specialist anesthetist trained in lung ultrasound with an M7series ultrasound machine (Shenzhen Myriad Biomedical Electronics Co., Ltd.). A 4–10 MHz line array probe was selected and the lung ultrasound was performed in the supine and lateral positions as described by Acosta et al. [10]. The thorax was divided into upper and lower hemithoraxe by two short axes (1 cm above the level of the diaphragm and 1 cm above the nipple line) and each hemithorax was divided into six regions by six long axes (anterior–posterior median line, right and left anterior axillary line and right and left posterior axillary line), making a total of 12 regions (Fig. 1). The probe was placed parallel to the intercostal space and scanned these 12 areas from anterior to posterior, top to bottom, and left to right, focusing on the signs of atelectasis and bronchial air-filled fluid signs.

**Fig. 2:**
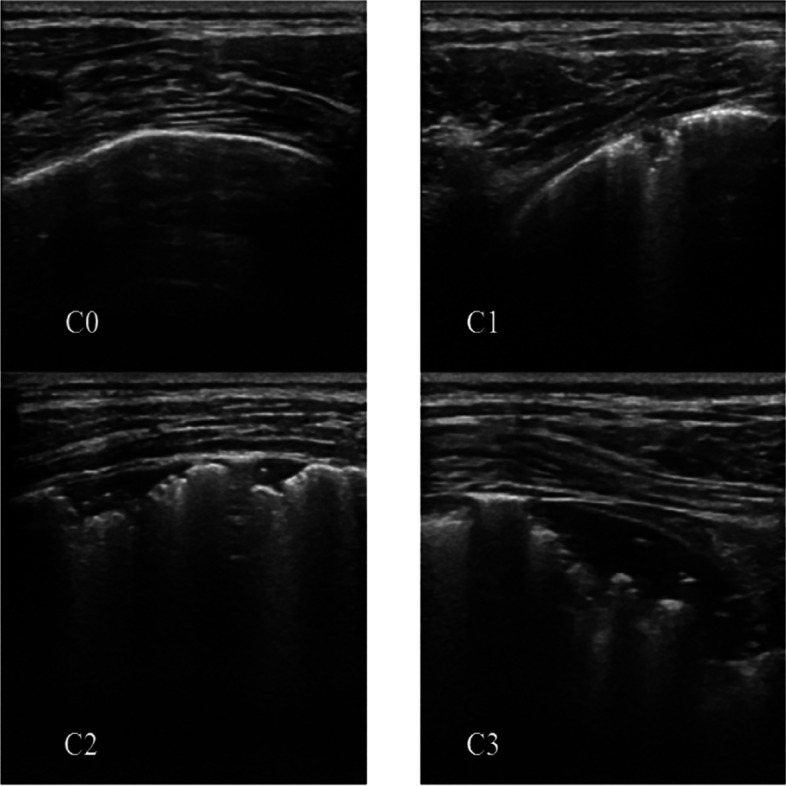
Ultrasound images of pulmonary atelectasis and grading of atelectasis

Atelectasis was divided into four grades under lung ultrasound (Fig. 2), which were assigned a score of 0–3 depending on the grade. 0: no atelectasis, 1. minimal pleural depression, 2. small regional solidity, 3. several small regional solidities fused into a large pulmonary solidity with visible signs of bronchial air-filled fluid. The most severe consolidation score in each region was used as the region’s atelectasis score, and the atelectasis scores in each region were summed to obtain the child’s atelectasis score [11].

### Outcome variables

The primary outcomes were the incidence of postoperative atelectasis (Because grade 1 atelectasis is easy to recover, only patients with grade 2 or higher atelectasis were included in the calculation of the incidence of atelectasis) and the postoperative atelectasis score. Secondary outcomes were baseline data, duration of anesthesia, duration of surgery, vital signs at three-time points including after insertion of the laryngeal mask, intraoperative (10 min after the start of the operation), and before the removal of the laryngeal mask. In addition, whether encounter complications such as reflux aspiration etc. were also recorded. Vital signs mainly including respiratory rate, heart rate, blood pressure, PetCO_2_ and exhaled tidal volume etc..

### Study sample size and statistical analysis

Our sample size was calculated using data obtained from pre-test results, the incidence of atelectasis in the spontaneous breathing group was approximately 90% and in the mechanical ventilation group was 40%. The sample size was calculated using G Power software, the require sample size was at least 22 patients in each group, with a set α error of 0.05, a power of 0.9. And a 10% loss to follow-up was also considered. Therefore, we enrolled 23 patients in each group.

SPSS 23.0 software was used for analysis. Normally distributed measurement data were expressed as mean ± standard deviation and a t-test for independent samples was used for comparison between groups. Skewed distribution data were expressed as median (quartile [full range]) and a non-parametric test was used for comparison between groups. Quantitative data were compared using the *x*^*2*^ test or Fisher’s exact probability method. *p*-values < 0.05 were considered statistically significant.

## Results

**Fig. 3:**
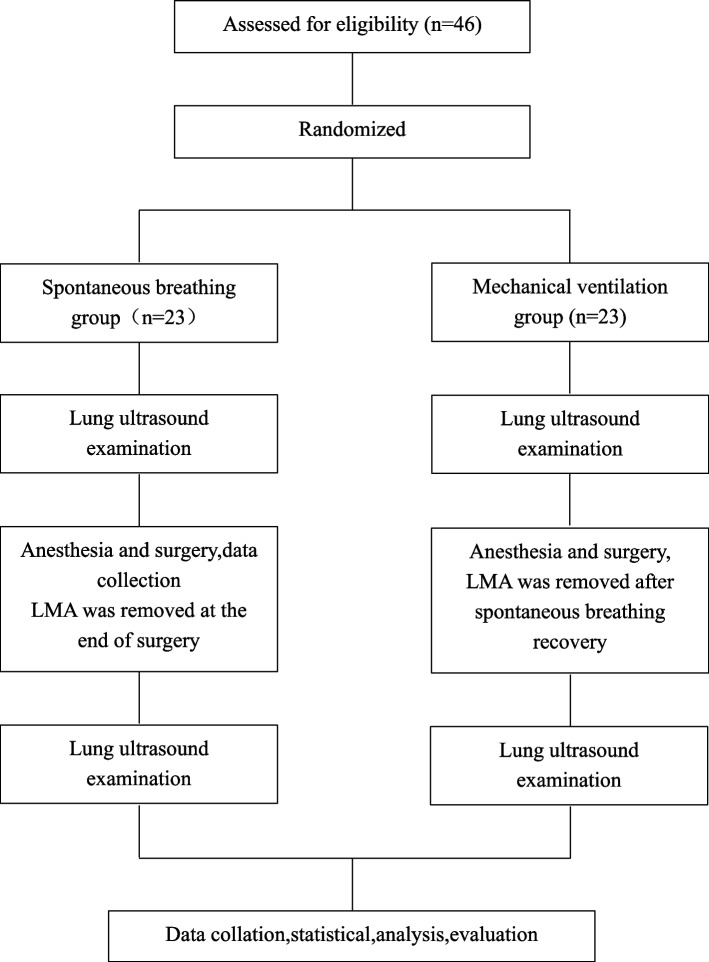
Consolidated standards of reporting trials flow diagram of patients included in the study

A total of 46 patients were included in both groups and no patients were excluded after inclusion in the trial (Fig. 3). In the first lung ultrasound examination, no atelectasis was found. In the second lung ultrasound examination, the incidence of atelectasis in the spontaneous breathing group (S Group, *n* = 23) was 91.30%, and in the mechanical ventilation group (M Group, *n* = 23) was 39.13%. There was a significant difference between the two groups, *P* = 0.001 (Table 1). The baseline information of the children in both groups were presented in Table 2 and no statistical difference was seen between the two groups (*P* > 0.05).

**Table 1:** Incidence of postoperative pulmonary atelectasis in children

	S Group (*N* = 23)	M Group (*N* = 23)	Chi-square value	*P*-value
Pulmonary atelectasis, n (%)	21 (91.30%)	9 (39.13%)	11.596	0.001

The S Group refers to spontaneous breathing group, The M Group refers to mechanical ventilation group

**Table 2:** Baseline information of the children

Factors	S Group (*N* = 23)	M Group (*N* = 23)	*P*-value
Gender			
Male	19	18	0.710
Female	4	5	
Age (y)	5.26 ± 1.42	5.72 ± 1.44	0.287
Body weight (kg)	22.21 ± 6.33	24.61 ± 8.00	0.265
Height (cm)	115.30 ± 12.51	119.61 ± 13.75	0.273
Anesthesia time (min)	33.65 ± 15.78	34.22 ± 19.49	0.914
Operating time (min)	24.09 ± 14.52	25.65 ± 17.97	0.747

The S Group refers to spontaneous breathing group, The M Group refers to mechanical ventilation group

The vital signs of the children were presented in Table 3. No statistically significant differences were seen in the vital signs of the children at the three-time points of after insertion of the Laryngeal mask (LMA), intraoperative, and before removal of the LMA (*p* > 0.05).

**Table 3:** Vital signs of the child

	S Group (*N* = 23)	M Group (*N* = 23)	*P*-value
After insertion of the LMA			
SpO_2_ (%)	99.52 ± 0.59	99.65 ± 0.57	0.452
HR (times/min)	115.83 ± 24.99	114.13 ± 17.98	0.793
MAP (mmHg)	69.96 ± 15.56	68.87 ± 8.79	0.772
RR (times/min)	18.43 ± 5.83	20.00 ± 0.00	0.205
Intraoperative			
SpO_2_ (%)	99.04 ± 1.49	99.65 ± 0.64	0.079
HR (times/min)	122.70 ± 22.90	121.87 ± 16.74	0.890
MAP (mmHg)	73.43 ± 15.43	74.65 ± 17.54	0.804
RR (times/min)	19.04 ± 6.49	20.00 ± 0.00	0.483
Before removal of the LMA			
SpO_2_ (%)	99.09 ± 1.08	99.48 ± 0.59	0.136
HR (times/min)	125.78 ± 22.35	117.00 ± 13.15	0.112
MAP (mmHg)	70.39 ± 14.89	74.30 ± 16.55	0.404
RR (times/min)	20.65 ± 6.18	20.00 ± 0.00	0.616

The S Group refers to spontaneous breathing group, The M Group refers to mechanical ventilation group, *LMA* Laryngeal mask

**Fig. 4:**
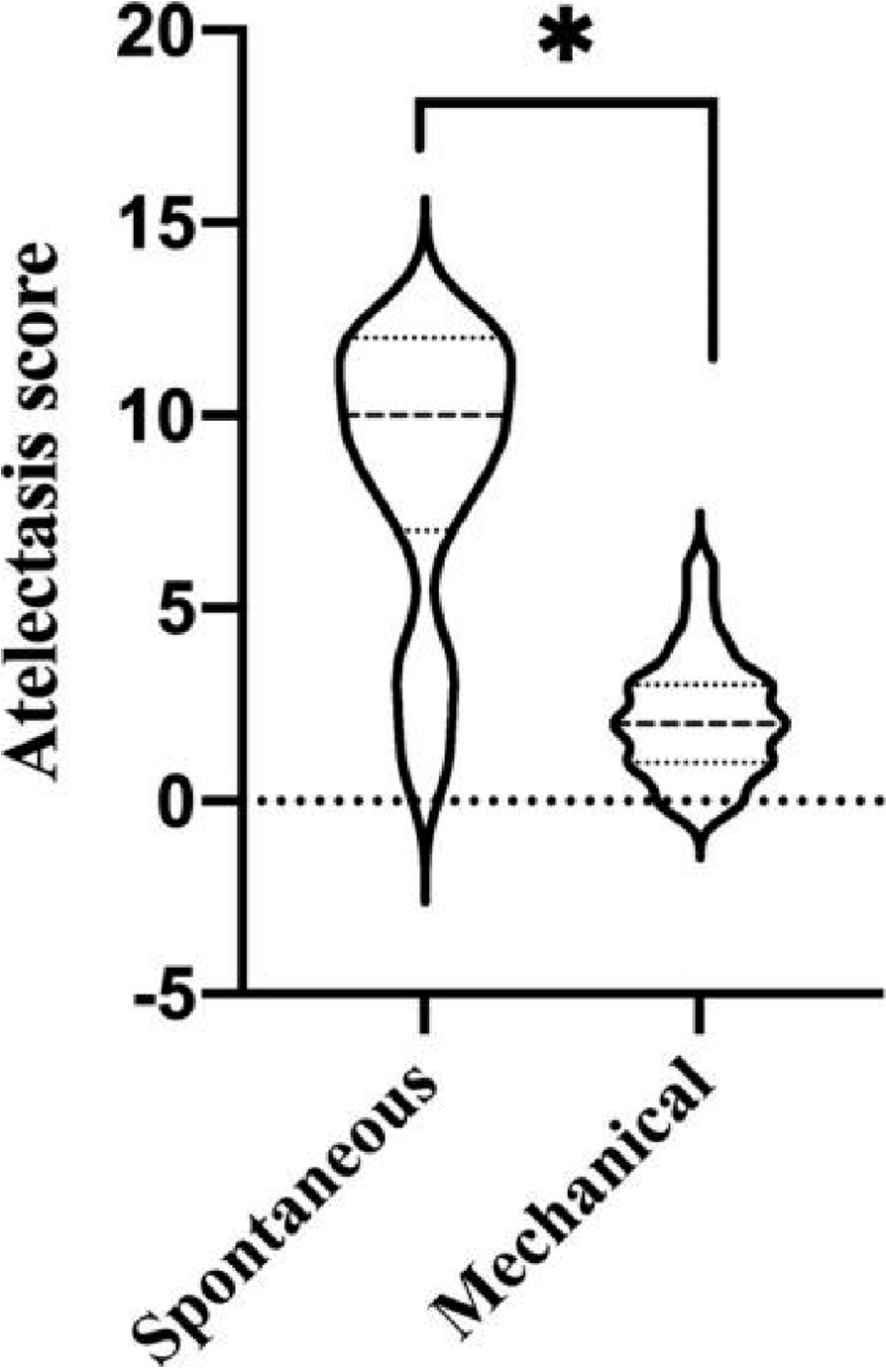
Patient’s post-operative atelectasis score

The exhaled tidal volume was significantly higher in the mechanical ventilation group than that in the spontaneous breathing group at the first two moments, and there is no difference between the S Group (before removal of laryngeal mask) and the M group (after spontaneous breathing recovered) at the third moment (Table 4). The end-expiratory carbon dioxide was significantly lower in the mechanical ventilation group than that in the spontaneous breathing group (*P* < 0.05) (Table 5). No pulmonary atelectasis occurred in either group on preoperative lung ultrasound examination, and in second lung ultrasound examination, the score of postoperative pulmonary atelectasis in the mechanical ventilation group 2(1–3[0–6]) was significantly lower than that in the spontaneous breathing group 10(7–12[1–12]) (*p* < 0.05) (Fig. 4).

**Table 4:** Exhaled tidal volume of the child

	S Group (*N* = 23)	M Group (*N* = 23)	*P*-value
After insertion of the LMA (ml/kg)	5.23 ± 2.39	8.04 ± 0.50	< 0.001
Intraoperative (ml/kg)	5.18 ± 2.04	7.80 ± 0.62	< 0.001
Before removal of the LMA (ml/kg)^a^	5.46 ± 2.04	6.22 ± 1.15	0.129

The S Group refers to spontaneous breathing group, The M Group refers to mechanical ventilation group, *LMA* Laryngeal mask

^a^S Group tidal volume: before the removal of laryngeal mask, M Group tidal volume: after spontaneous breathing recovery

**Table 5:** End-tidal carbon dioxide in children

	S Group (*N* = 23)	M Group (*N* = 23)	*P*-value
After insertion of the LMA (mmHg)	47.43 ± 5.51	39.87 ± 5.60	< 0.001
Intraoperative (mmHg)	51.83 ± 7.43	36.39 ± 5.34	< 0.001
Before removal of the LMA (mmHg)	50.83 ± 8.67	36.43 ± 5.14	< 0.001

The S Group refers to spontaneous breathing group, The M Group refers to mechanical ventilation group, *LMA* Laryngeal mask

## Discussion

Children undergoing day surgery have a short hospital stay, usually within 24 h, and atelectasis remains in the body for a long time after surgery [12, 13], which increases the risk of hypoxemia and pulmonary infection, and affects the safety of patients during the perioperative period. Therefore, day surgery requires a relatively high-quality anesthesia, and it is particularly important to reduce the incidence of postoperative atelectasis in children undergoing day surgery.

In this study, the incidence of pulmonary atelectasis was 91.30% in the spontaneous breathing group and was 39.13% in the mechanical ventilation group, which indicating that the incidence of pulmonary atelectasis in patients undergoing laryngeal mask general anesthesia is high, it means that we need to pay more attention to children who underwent day surgery. In this trial, we found that the atelectasis score in the mechanical ventilation group was significantly lower than that in the spontaneous breathing group, This results indicate that the use of mechanical ventilation in PCV mode can reduce the incidence of postoperative pulmonary atelectasis in patients undergoing laryngeal mask general anesthesia, which may mainly due to the inconsistency of respiratory physiology between spontaneously breathing patients and mechanically ventilated patients.

The formation of perioperative atelectasis is still not fully understood and there are three mechanisms may explain the formation of atelectasis [14], including compressional atelectasis, absorptive atelectasis, and atelectasis due to impaired alveolar surface-active substances. When the functional residual air volume (FRC) of the lung decreases, the terminal fine bronchi in part of the lung are occluded and the gas in the alveolar tissue distal to the occlusion diffuses into the pulmonary circulation through the capillaries, while the proximal occluded alveoli are not replenished with fresh gas, the alveoli gradually atrophy and atelectasis develop. It has been reported [15] that anesthetic drugs, such as propofol and sufentanil, disrupt the balance between the respiratory muscles. After the injection of anesthetic drugs, the patient’s airway dilator muscles become dysfunctional, causing the airway tend to close, and then absorption atelectasis developed. This process is exacerbated by the increased negative intrathoracic pressure during inspiration in spontaneously breathing patients. However the mechanism was opposite in the mechanical group, where the PCV mode exerts a set of positive pressures and the intra-airway pressure can be balanced with the extra-airway pressure to keep the airway open and prevent the development of atelectasis. In addition, at normal functional residual capacity, the inward alveolar retraction force is in equilibrium with the elastic expansion force of the chest wall, which does not usually change in patients without chest wall disease, and the inward alveolar retraction force is correlated with tidal volume [16]. In this study, we found that the exhaled tidal volume was significantly lower in the spontaneous breathing group than mechanical ventilation group, which was consistent with the results of Lim B et al. [6–9]. The children in the spontaneous breathing group were more susceptible to atelectasis because they had smaller tidal volumes, which meant that their inward retraction forces were larger than those of the mechanical breathing group. As a result, the incidence of atelectasis and atelectasis scores in mechanical ventilation patients were significantly lower than those in spontaneous breathing group. In addition we find there were no significant difference in tidal volume before removing the laryngeal mask between spontaneous breathing group and mechanical ventilation group, but there were significant difference in introperative and after insertion of laryngeal mask. Furthermore Edmark L [17] demonstrated that the atelectasis formed several minutes after induction, So it means that provide a high tidal volume after induction can prevent the formation of atelectasis.

In this investigation, we discovered that tidal volume has a significant impact on PetCO_2,_ because the exhaled tidal volume was significantly higher in the mechanical ventilation group than spontaneous breathing group, and PetCO_2_ was significantly lower in the mechanical ventilation group than spontaneous breathing group. A previous clinical trial [18] showed that mechanical ventilation with laryngeal mask or spontaneous breathing did not affect the gas diffussion into the pulmonary circulation, whereas Increased carbon dioxide levels can lead to hypercapnia, acidosis, prolonged wake-up time and postoperative irritability etc., so in the perioperative care of patients receiving general anesthesia with a laryngeal mask, it is still crucial to be aware of the emergence of carbon dioxide accumulation. In this study, the driving pressure in the mechanical ventilation group was below 20 cmH_2_O, and no patients experienced complications such as reflux aspiration. Furthermore previous literature has reported [19] that laryngeal mask mechanical ventilation didn’t increase the risk of reflux aspiration, so mechanical ventilation was safe and effective in the perioperative management of patients undergoing laryngeal mask general anesthesia. Therefore, we recommend mechanical ventilation for airway management in patients who need a laryngeal mask general anesthesia for day surgery.

Atelectasis is a common postoperative pulmonary complication, which can increase intrapulmonary shunt, lead to hypoxemia, increase the risk of postoperative pneumonia in children, and affect the safety of children during the perioperative period. At present, there are no large-sample, multi-center clinical studies explaining whether it’s necessary to treatment postoperative atelectasis in individuals who have healthy preoperative lungs. Zeng C [20] reported that the presence of atelectasis can increase respiratory resistance and reduce lung compliance, and this effect persists even after the patient’s atelectasis recovers. According to Nakos G. et al. [21], the phospholipid content of surfactant in the region of atelectasis was decreased and remained low even after lung reopening. This decrease in phospholipid content harmed the lung’s capacity to fight infection and raised the likelihood of lung infection following surgery. Regarding the retention time of postoperative atelectasis in the body, Strandberg A [12] et al. used CT scans for patients undergoing hernia surgery and found that 90% of the patients had atelectasis within 1 h after surgery, and atelectasis is still present in 50% of patients after 24 h after surgery. A retrospective analysis [13] reviewed the postoperative CT imaging features of 944 patients undergoing non-cardiac surgery and found that 539 (57%) patients developed pulmonary atelectasis, of which 57 of these patients showing little sign of improvement by day 3 postoperative, which indicating that postoperative pulmonary atelectasis persists for a long time in the body. In addition, the formation of atelectasis leads to an increase in inflammatory factors such as IL-1, IL-6, IL-8 and tumor necrosis factor in the body, raising the risk of lung injury, and the amount of inflammatory factors were correlated with the duration of atelectasis in the body [22, 23]. These all suggest that atelectasis has an immediate and severe impact on patients undergoing general anesthesia, and that it lasts a long time in the body. As a result, it is especially crucial to reduce the incidence of atelectasis in the perioperative period.

Laryngeal mask is a common intraoperative airway management tool for patients undergoing day surgery. It can be used for either spontaneous breathing or mechanical ventilation. Retention of spontaneous breathing can reduce reflux aspiration, avoid man–machine confrontation, and reduce the use of muscle relaxants, so it has become a commonly used respiratory management method in clinical practice. In the UK, 60% of patients were managed by the laryngeal mask with spontaneous breathing [24]. Fesseau et al. [25] and others found through a questionnaire survey of 232 anesthesiologists that the laryngeal mask retaining spontaneous breathing was the most commonly used breathing management method, but the laryngeal mask retaining spontaneous breathing may also lead to hypoventilation, atelectasis, hypercapnia, etc., there were only a few literatures [6–9] comparing the advantages and disadvantages of these two ventilation methods by observing the respiratory parameters of patients during the perioperative period, they found that the operation can be completed safely and effectively by these two types of breathing management. However, spontaneous breathing can lead to adverse events such as low tidal volume and hypercapnia, so it’s worth further discussion on which respiratory management method is better. Now, no researchers have contrasted the benefits and drawbacks of the two ventilation strategies from the standpoint of atelectasis. Therefore, it is creative to explain which ventilation strategy is preferable from the perspective of atelectasis.

There are several limitations in this study. First, this is a single center, small sample study. so our findings need to be validated in a multicentre study. Second, day surgery patients have a short hospital stay and lack of observation on the long-term effects of atelectasis.

The use of mechanical ventilation in patients with laryngeal mask general anesthesia can reduce the incidence and severity of atelectasis, prevent the accumulation of carbon dioxide, and we didn’t encounter any complications such as reflux and aspiration in this study. Therefore, we recommend that children who need a laryngeal mask for day surgery using mechanical ventilation for airway management.

## Data Availability

The datasets used and/or analysed during the current study available from the corresponding author on reasonable request.

## References

[1] Lutterbey G, Wattjes MP, Doerr D, Fischer NJ, Gieseke J, Schild HH (2007). Atelectasis in children undergoing either propofol infusion or positive pressure ventilation anesthesia for magnetic resonance imaging. Paediatr Anaesth.

[2] van Kaam AH, Lachmann RA, Herting E (2004). Reducing atelectasis attenuates bacterial growth and translocation in experimental pneumonia. Am J Respir Crit Care Med.

[3] Liu G-L, Zhang J-M, Zheng T-H (2014). The use of laryngeal mask in short neonatal surgery. J Clin Anaesth.

[4] Wu ZY, Cha BJ, Wang YS (2013). Effectiveness of nasopharyngeal airway-mask autonomic breathing with propofol-fentanyl-sevoflurane combined with sedation for general anesthesia in pediatric short and minor surgery: comparison with laryngeal mask autonomic breathing. Chin J Anesthesiol.

[5] Zhou RL, Hang YN (2006). Clinical application of third-generation laryngeal mask [J]. J Clin Anaesth.

[6] Dhar R, Sofi K, Mir SA, Jehangir M, Wazir M (2021). Comparison of spontaneous ventilation, pressure control ventilation and pressure support ventilation in pediatric patients undergoing infraumbilical surgery using proseal laryngeal mask airway. Anesth Essays Res..

[7] Templeton TW, Hoke LK, Yaung J, Aschenbrenner CA, Rose DM, Templeton LB, Bryan YF (2016). Comparing 3 ventilation modalities by measuring several respiratory parameters using the ProSeal laryngeal mask airway in children. J Clin Anesth.

[8] Lim B, Pawar D, Ng O (2012). Pressure support ventilation vs spontaneous ventilation via ProSeal laryngeal mask airway in pediatric patients undergoing. Paediatr Anaesth.

[9] Jeong H, Tanatporn P, Ahn HJ, Yang M, Kim JA, Yeo H, Kim W (2021). Pressure support versus spontaneous ventilation during anesthetic emergence-effect on postoperative atelectasis: a randomized controlled trial. Anesthesiology.

[10] Acosta CM, Maidana GA, Jacovitti D (2014). Accuracy of transthoracic lung ultrasound for diagnosing anesthesia-induced atelectasis in children. Anesthesiology.

[11] Song IK, Kim EH, Lee JH (2017). Effects of an alveolar recruitment manoeuvre guided by lung ultrasound on anaesthesia-induced atelectasis in infants: a randomised, controlled, trial. Anaesthesia.

[12] Strandberg A, Tokics L, Brismar B, Lundquist H, Hedenstierna G (1986). Atelectasis during anaesthesia and in the postoperative period. Acta Anaesthesiol Scand.

[13] Mavros MN, Velmahos GC, Falagas ME (2011). Atelectasis as a cause of postoperative fever. Where is the clinical evidence?. Chest.

[14] Duggan M, Kavanagh BP (2007). Atelectasis in the perioperative patient. Curr Opin Anaesthesiol.

[15] Sasaki N, Meyer MJ, Eikermann M (2013). Postoperative respiratory muscle dysfunction: pathophysiology and preventive strategies. Anesthesiology.

[16] Hess DR (2014). Respiratory mechanics in mechanically ventilated patients. Respir Care.

[17] Edmark L, Kostova-Aherdan K, Enlund M, Hedenstierna G (2003). Optimal oxygen concentration during induction of general anesthesia. Anesthesiology.

[18] Gan, Linguang, Chen, Junping (2011). Effect of different ventilation modes on lung function during SLIPA mask ventilation for short and minor surgery. Chin Med J.

[19] Ye Q, Wu D, Fang W, Wong GTC, Lu Y (2020). Comparison of gastric insufflation using LMA-supreme and I-gel versus tracheal intubation in laparoscopic gynecological surgery by ultrasound: a randomized observational trial. BMC Anesthesiol.

[20] Zeng C, Lagier D, Lee JW, Vidal Melo MF (2022). Perioperative pulmonary atelectasis: part I. Biol Mech Anesthesiol.

[21] Nakos G, Tsangaris H, Liokatis S, Kitsiouli E, Lekka ME (2003). Ventilator-associated pneumonia and atelectasis: evaluation through bronchoalveolar lavage. Intensive Care Med.

[22] Komatsu Y, Yamamoto H, Tsushima K, Furuya S, Yoshikawa S, Yasuo M, Kubo K, Yamazaki Y, Hasegawa J, Eguchi T, Kondo R, Yoshida K, Koizumi T (2012). Increased interleukin-8 in epithelial lining fluid of collapsed lungs during one-lung ventilation for thoracotomy. Inflammation.

[23] Sugasawa Y, Yamaguchi K, Kumakura S, Murakami T, Suzuki K, Nagaoka I, Inada E (2012). Effects of sevoflurane and propofol on pulmonary inflammatory responses during lung resection. J Anesth..

[24] Verghese C, Brimacombe JR (1996). Survey of laryngeal mask airway usage in 11,910 patients: safety and efficacy for conventional and nonconventional usage. Anesth Analg.

[25] Fesseau R, Alacoque X, Larcher C, Morel L, Lepage B, Kern D (2014). An ADARPEF survey on respiratory management in pediatric anesthesia. Paediatr Anaesth.

